# Evaluating the potential impact of proton carriers on syntrophic propionate oxidation

**DOI:** 10.1038/srep18364

**Published:** 2015-12-16

**Authors:** Natacha M. S. Juste-Poinapen, Mark S. Turner, Korneel Rabaey, Bernardino Virdis, Damien J. Batstone

**Affiliations:** 1 The University of Queensland, Advanced Water Management Centre, St. Lucia, QLD 4072, Australia; 2 The University of Queensland, School of Agriculture and Food Sciences, St. Lucia, QLD 4072, Australia; 3 Ghent University, Laboratory of Microbial Ecology and Technology (LabMET), 9000 Ghent, Belgium; 4 The University of Queensland, Centre for Microbial Electrochemical Systems, St. Lucia, QLD 4072, Australia

## Abstract

Anaerobic propionic acid degradation relies on interspecies electron transfer (IET) between propionate oxidisers and electron acceptor microorganisms, via either molecular hydrogen, formate or direct transfers. We evaluated the possibility of stimulating direct IET, hence enhancing propionate oxidation, by increasing availability of proton carriers to decrease solution resistance and reduce pH gradients. Phosphate was used as a proton carrying anion, and chloride as control ion together with potassium as counter ion. Propionic acid consumption in anaerobic granules was assessed in a square factorial design with ratios (1:0, 2:1, 1:1, 1:2 and 0:1) of total phosphate (TP) to Cl^−^, at 1X, 10X, and 30X native conductivity (1.5 mS.cm^−1^). Maximum specific uptake rate, half saturation, and time delay were estimated using model-based analysis. Community profiles were analysed by fluorescent *in situ* hybridisation and 16S rRNA gene pyrosequencing. The strongest performance was at balanced (1:1) ratios at 10X conductivity where presumptive propionate oxidisers namely *Syntrophobacter* and *Candidatus Cloacamonas* were more abundant. There was a shift from *Methanobacteriales* at high phosphate, to *Methanosaeta* at low TP:Cl ratios and low conductivity. A lack of response to TP, and low percentage of presumptive electroactive organisms suggested that DIET was not favoured under the current experimental conditions.

In a methanogenic environment, in the absence of an external electron acceptor such as nitrate or sulfate, anaerobic oxidation of organic acids to acetate and hydrogen (H_2_) is only thermodynamically feasible where microorganisms associate to allow electron sinking to a methanogen^1^. This process is known as syntrophy and involves the transfer of electrons from one microorganism (electron donor) to the other (electron acceptor) by interspecies electron transfer (IET)^2^,^3^.

Propionate is a key substrate oxidised via syntrophy under methanogenic conditions, with two distinct reactions combining to generate catabolic energy necessary for the survival of the partners involved. Known syntrophic propionate oxidisers include *Syntrophobacter wolinii*^4^, *Syntrophobacter sulfatireducens*^5^, *Syntrophobacter fumaroxidans*^6^, *Pelotomaculum schinkii*^7^ and *Smithella propionica*^8^. Strain *Candidatus Cloacamonas acidaminovorans* has also been linked to syntrophic propionate oxidation^9^,^10^. The net microbial reaction is oxidation of propionate to acetate and hydrogen with energy generated through substrate level phosphorylation on the propionyl-CoA (Eq. 1)^1^,^11^,12.


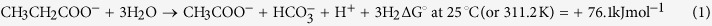


An alternative reaction in *Smithella* sp involves dismutation of 2 propionate molecules with final products butyrate and acetate in a fermentation reaction (Eq. 2):


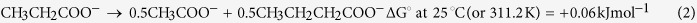


The butyrate is further oxidised to acetate and H_2_^13^,^14^, which results in a net equation equivalent to Eq 1. In order to enable oxidation reactions in either case, electrons are utilised by methanogens to reduce carbon dioxide (CO_2_) to methane (CH_4_). Hydrogenotrophic methanogenesis reduces CO_2_ to CH_4_. Most H_2_ utilising methanogens do not have cytochromes^15^,^16^, which suggests that energy is generated mostly through a sodium (cation) motive force (SMF)^17^.

The most commonly proposed mechanism for electron transfer is mediated IET (MIET) with H_2_ and/or formate as the electron carriers^18^,^19^. However other oxidative organisms fulfilling similar metabolic niches on other substrates have been observed to engage in direct electron transfer as detailed below.

Co-cultures of *Geobacter* sp engineered to study interspecies H_2_ transfer have been shown to engage in direct IET instead which was metabolically more favourable, with growth observed within 21 days of incubation instead of 7 months^20^. DIET, via membrane-bound proteins (OmcS)^21^ or through conductive pili, was observed in environmental samples during the degradation of ethanol where *Geobacter* sp, capable of DIET, were the dominant oxidisers and *Methanosaeta* sp the electron acceptor microorganism^21^,^22^.

DIET partnerships have not been widely observed in natural communities possibly because existing culture conditions favour MIET and difficulty in transferring DIET engaged cultures^23^. Advantages of DIET vs MIET are that molecular diffusional limitations (and gradients) are avoided and translation of electrons to a molecular carrier are not required. However, as a potential limitation, DIET involves the direct transport of electrons between cells and also requires the migration of a charged ions to maintain electroneutrality^24^. Charge can be transferred either as protons, or as saline anions and cations, and generally, the latter (anions and cations) will dominate, due to the very low concentrations of protons at neutral pH conditions. Migration of saline ions will result in a pH gradient, with a low pH at the DIET oxidiser, and high pH at the DIET reducer. This can result in inhibition by pH changes and decreased thermodynamic viability, due to charge accumulation. Under such conditions, increasing proton mobility outside the cells would increase proton availability.

Buffers increase proton transport rate by molecular diffusion of the buffer, particularly in its associated form (allowing pH gradient neutralisation). In addition, the presence of the buffer will mitigate pH gradients caused by transport of saline ions due to the buffering effect of the weak base. As early as 1979, Engasser and Wilhem-Despres proved the ability of buffers, at a higher concentration, to shuttle protons, which could help improve processes such as ion exchange^25^. Torres *et al.* (2008) have shown that phosphate buffer facilitates proton transfer from anode-respiring electroactive biofilm to bulk liquid in single chamber microbial fuel cells, translating into higher current densities, while Nagarajan *et al.* (2013) have predicted a direct influence of proton mobility on the energetic efficiency of DIET. It was noted that total salinity will also enhance DIET, through a decrease in solution resistance, but this will also subject the microbial community to saline inhibition^24^,^26^,^27^.

Therefore, the presence of buffers, particularly phosphate, which have pKa values near circumneutral values (6–8), should enhance DIET, by reducing pH gradients, and enhancing proton mobility. To test this hypothesis (that DIET and hence activity is promoted by proton motility), this paper evaluates propionate utilisation capacity under varying TP:Cl^−^ (TP = total phosphate) ratio (5 levels) and varying total conductivity (3 levels) in a square factorial design. Potassium was used as counter ion to minimise inhibition effects. Experiments were designed to assess whether either solution salinity or proton motility separately have an influence on propionate utilisation rate, and by inference, direct interspecies electron transfer. The inoculum used consisted of anaerobic granules from anaerobic digester treating brewery waste, which are known to be dominated by IET^14^.

## Results

Specific Methane Activity tests were initially carried out on intact anaerobic granules to establish the influence of crushing the granules on the overall experiment (Fig. S1). A small delay was noted at the onset of the experiment when the physical property was modified, but the exponential curves are comparable.

### Chemical Analysis

**Figure 1:**
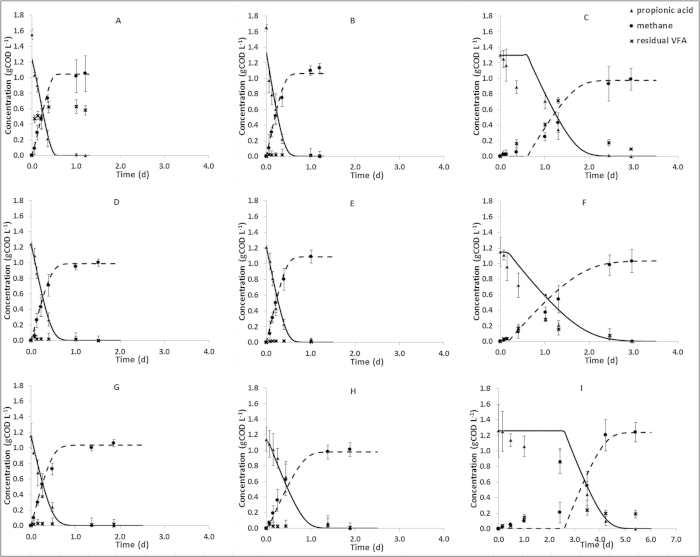
Measured cumulative methane production (•), propionic acid concentrations (▲) and net non-propionate organic acids (x) together with model curves (−) for assays at ratio and conductivity 1:0 1X (**A**), 1:0 10X (**B**), 1:0 30X (**C**), 1:1 1X (**D**), 1:1 10X (**E**), 1:1 30X (**F**), 0:1 1X (**G**), 0:1 10X (**H**) and 0:1 30X (**I**). Error bars represent 95% confidence in means based on triplicate analyses. The six additional tests (1:2 and 2:1 1X, 10X, 30X) are provided in Fig. S2. Error bars represent 95% confidence based on triplicate analyses.

Biogas and VFA analysis showed concomitant production of methane gas with propionic acid utilisation (Fig. 1A–I). Acetate, butyrate, valerate and hexanoate made up the VFA end-products and were usually maintained at low residual levels (<0.2 gCOD L^−1^) except in the two first time point samples, with 0.5 gCOD L^−1^ (3:1:1 C_2_:C_4_:C_5_) seen in 1X conductivity and 100% TP, 0.9 gCOD L^−1^ (4:1:3 C_2_:C_4_:C_5_) seen in 30X conductivity and 100% Cl^−^.

When NaCl was used as the main chloride salt at similar concentration to KCl (Table S1), increasing conductivity to 30X resulted in a low methane yield with no further increase after 3.5 days of incubation (Fig. S2). In contrast, as shown in Fig. 1-C,F,I with KCl, experiments carried out at 30X conductivity experienced a delay or lag phase, being higher at high Cl^−^ levels and recorded as 0.5 (±0.1) days, 0.23 (±0.08) days, 0.22 ± (0.08) days, 0.4 (±0.1) days and 2.6 (±0.1) days, for the ratio of 1:0, 2:1, 1:1, 1:2 and 0:1 respectively.

Since increase in biomass has previously been related to increase in microbial growth^28^, the changes in VS were used as a quantitative assumption for any increase or decrease in the microbial population (Table S2). With an initial VS value of 2.27 ± 0.04 g L^−1^, an overall increase in biomass was noted after SMA in most experiment.

**Figure 2:**
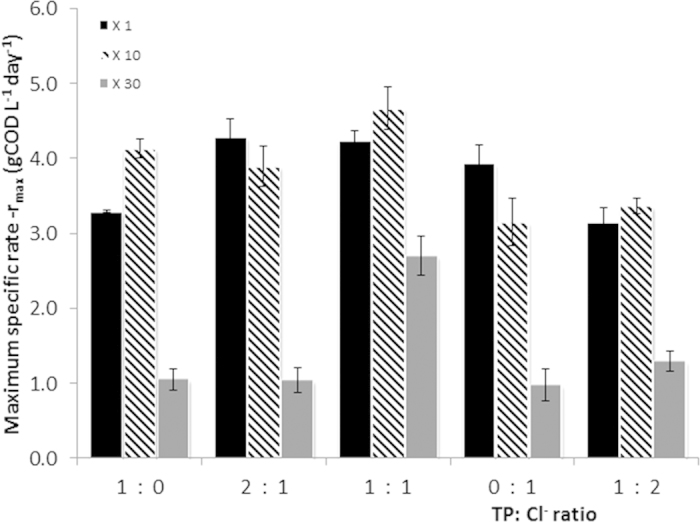
Comparison of the changes in the maximum specific rate (r_max_) in the different batch tests run at different ratio of TP:Cl^−^ at 1X (■), 10X (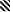) and 30X (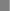) conductivities. Error bars represent 95% confidence in the estimated parameters.

Figure 2 reports the maximum specific rate (r_max_) of methane production for each assay. The r_max_ values increased with changes in TP:Cl^−^ ratio, with the highest values of 4.24 (±0.27) gCOD L^−1^ day^−1^ and 4.28 (±0.25) gCOD L^−1^ day^−1^ found with ratios of 1:1 and 2:1 respectively. The highest r_max_ of all the tested batches was at 10X conductivity with a 1:1 ratio, at 4.67 (±0.28) gCOD L^−1^ day^−1^. Lower values were recorded at higher Cl^−^ concentration, for samples at 1:2 and 0:1 ratio. At 30X conductivity, the r_max_ value decreased significantly across all the samples, with the highest value at only 2.70 (±0.32) gCOD L^−1^ day^−1^ with equimolar PO_4_^3−^ and Cl^−^ (Fig. 2).

According to correlation analysis the variation in r_max_ across treatments was positively correlated with variability in conductivity (*r* = −0.809, *p* = 0.008). High conductivities were also significantly correlated with time delay (*r* = 0.774, *p* = 0.001) and had a significant impact on changes in biomass (*r* = 0.543, *p* = 0.036) with, also, a negative correlation with the rate of the reaction (*r* = 0.682, *p* = 0.043).

### Microbial community response

**Figure 3:**
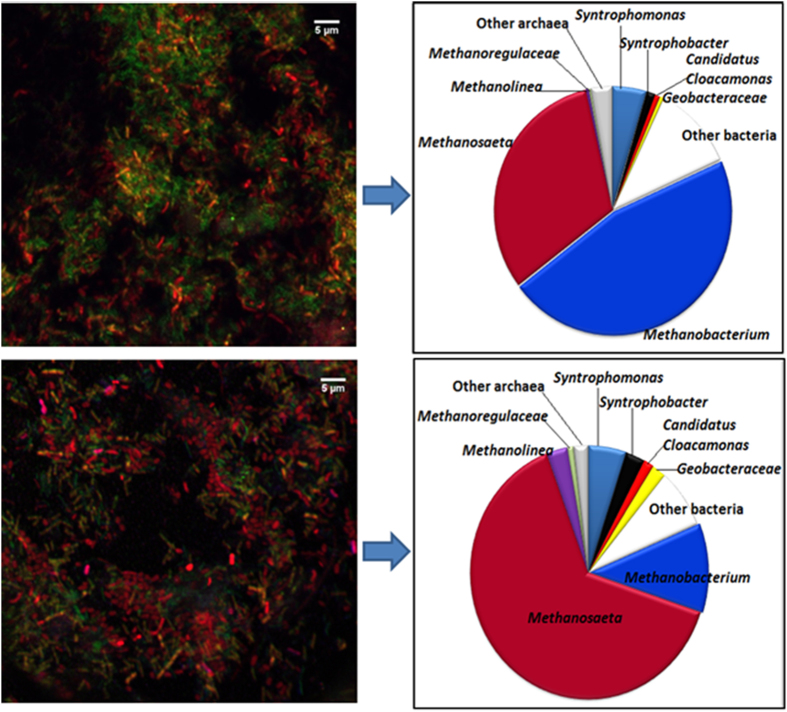
Complementary FISH and 16S rRNA gene pyrosequencing analysis of the microbial population in the original anaerobic granule (**A**) and assays at 1:1 ratio of TP:Cl^-^ (**B**) analysed at the end of the experiment. It includes FISH images with *Methanosaeta* sp in orange/yellow (MX825 and ARC915), all other archaeas in green (ARC915), *Syntrophobacter* sp in magenta (SYN835 & EUBmix) and all other bacteria in red (EUBmix), and corresponding percentage relative abundance of the different microorganisms present is illustrated in the pie charts.

**Figure 4:**
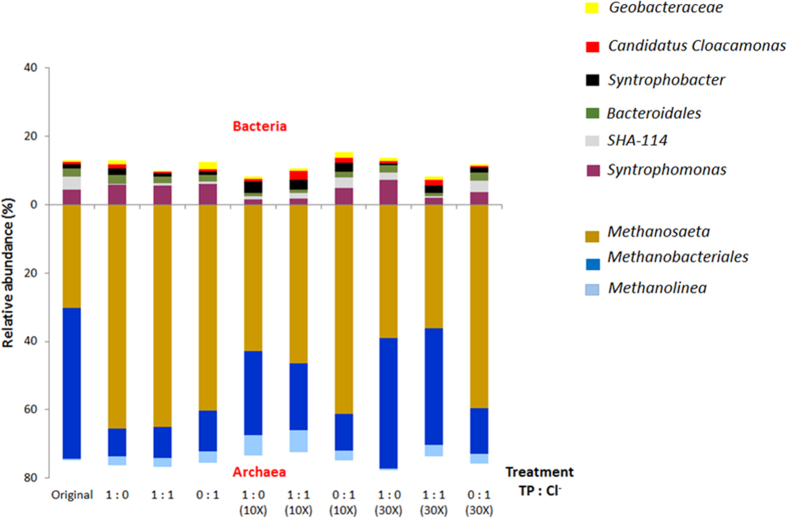
Microbial community profiles of anaerobic granules under different conductivity and TP:Cl^−^ ratio, including percentage relative abundances of the dominant taxonomic archaeal and bacterial groups mostly influenced by the current experimental conditions (>1.5%).

Both FISH and pyrosequencing performed on the initial inoculum showed that bacteria were relatively low in abundance, compared to the archaea (Fig. 3). Pyrosequencing results initially identified *Syntrophomonas* (4.6%), *Bacteroidales* (3.9%) and the class of SHA-114 (2.6%) as the most prevalent bacteria in the community dominated by three main genera of archaea as being *Methanobacterium*, *Methanosaeta and Methanolinea* (Fig. 4 & Table S3).

**Figure 5:**
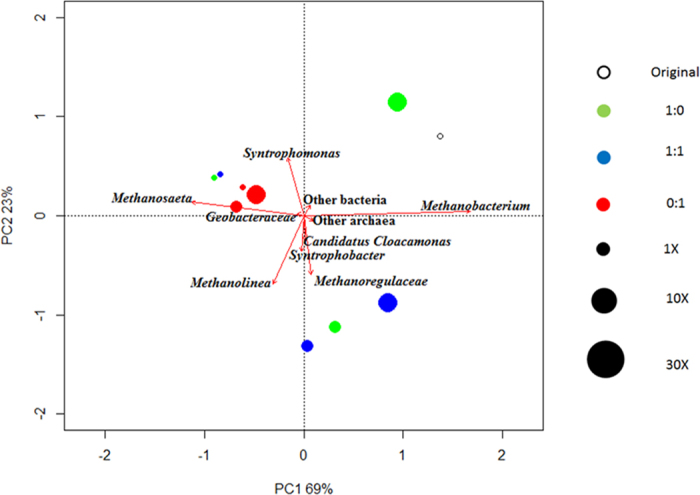
Principal Component analysis of the microbial population under different conductivity and TP: Cl^−^ ratio, including major archaea *Methanobacterium*, *Methanosaeta*, *Methanolinea*, propionate oxidisers *Syntrophobacter* and *Candidatus Cloacamonas* as well as the *Geobacteraceae* family capable of DIET and the originally dominant bacteria, *Syntrophomonas*.

This work focused specifically on presumptive syntrophic propionate oxidisers. While, very few cells from the *Syntrophobacter* species were identified with FISH (determined with mixed Syn 835 with EUB mix-Cy3 in Fig. 3), the pyrosequencing analysis identified two known propionate oxidisers belonging to the *Syntrophobacter* genus and *Candidatus Cloacamonas* genus initially at 1.3% and 0.7% relative abundance, respectively. As seen in Fig. 4, while there were no obvious changes in the abundance of *Syntrophobacter* at 1X conductivity, an increase was observed at 10X conductivity with percentages of 3.1%, 3.6% and 2.6% at 1:0, 1:1 and 0:1 TP to Cl^−^ ratio respectively. In comparison, *Candidatus Cloacamonas* was more susceptible to conditions with TP only and Cl^−^ only. At higher conductivities (10X and 30X), presumptive propionate oxidisers (see above) were positively correlated with samples that had TP and Cl^−^ ratio of 1:1 as further confirmed by PCA across PC2 (Fig. 5).

The only bacteria known to participate in DIET belonged to the *Geobacteraceae* family and were originally in quite low abundance (0.6%), with an increase in relative abundance noted in assays at 1X conductivity and a more substantial increase of 2.13% with Cl^−^ only at 10X conductivity (Fig. 4).

Another feature demonstrated in Fig. 5 is that low conductivity samples, and high-conductivity chloride only samples cluster (negative on PC1), while higher conductivity samples with phosphate are more widely distributed and are generally positive in PC1. This is mainly driven by a shift from dominant OTU *Methanobacteriales* in the inoculum and high phosphate samples, towards dominant OTU *Methanosaeta* in low conductivity and low phosphate samples. Specifically, the population shift averaged 46% to 13% for *Methanobacteriales*, corresponding to an average increase for *Methanosaeta* from 30% to 60%. Variation in high-phosphate samples is mainly caused by a variation in bacterial community.

## Discussion

Propionic acid utilisation with consequent methane production is an indication of syntrophic IET. Kinetic parameters indicate that the best results were obtained with a balanced TP:Cl^-^ ratio in all conductivities tested and that activity decreased as the balance shifted to phosphate or chloride, particularly at higher conductivities.

Overall, correlation analysis indicates that conductivity is the major factor influencing the system, including changes in community profile (also related to availability of phosphate), delay (which is an indicator of short term adaptation), and rate. The correlation analysis tests for linear relationships and indicates no linear relationship between TP:Cl^−^ ratio and activity. However, results clearly suggest a non-linear relationship, with an optimum occurring at a balanced TP:Cl^−^ ratio, suggesting that the reaction is most effective in the presence of both anions.

Despite previous research showing that low pH conditions can have a negative impact during anaerobic oxidation of organics^29^,^30^, in our study the system managed to recover and this was attributed to the presence of both buffers and potassium ions (K^+^). Specifically, buffers mitigate pH decreases caused by accumulation of organic acids, while potassium (after adaptation) enables a degree of pH tolerance^31^.

Mechanistically, an increase in buffer and/or salt concentration, results in an accumulation of K^+^, in order to maintain cell turgor pressure^32^. At high conductivity, in the presence of Na^+^, inhibition is stronger if K^+^ is limiting. This is known as K^+^ starvation and is translated in initial growth, reaching a quick stationary phase as the level of K^+^ ions in the media decreases^32^,^33^, a condition shown in batches treated with NaCl (Fig S2).

A more diverse microbial population was observed in those treatments that resulted in a higher growth rate, namely at 1:0 and 1:1 TP to Cl^−^ ratio at 10X conductivity and 1:1 ratio at 30X conductivity. This was confirmed by both rarefaction measurement (Fig. S5) and the Shannon index (Table S4). Two populations of hydrogenotrophic methanogens belonging to the genus *Methanobacterium and Methanolinea* were identified. Our findings support the initial sensitivity of *Methanobacterium* species, particularly at higher Cl^−^ to TP ratio, over the acetoclastic *Methanosaeta* and H_2_ utilising *Methanolinea*, phylogenetically related to *Methanolinea tarda* (Fig. S3) of the *Methanoregulaceae* family, capable of accepting electrons from both H_2_ and formate for methane production^34^.

The two main propionate oxidisers identified belonged to the genus *Syntrophobacter* and *Candidatus Cloacamonas*, with both abundant at 10X conductivity and 1:1 TP to Cl^−^ ratio, where the highest r_max_ was recorded. So far, neither has been linked to DIET. A literature search in the genome of these species was carried out. Since the genome of *Syntrophobacter wolinii*, phylogenetically linked to our *Syntrophobacter* cluster (Fig. S4), is not yet available, *Syntrophobacter fumaroxidans*^35^ was used as the closest relative strain. While membrane-bound cytochrome involved in ion-translocating ferredoxin:NADH oxidoreductase (Sfum_2694–99) and other transmembrane cytochromes linked to proton transfer were characterised, no pili-type genes or cytochromes that could be linked to DIET was detected in the genome of *Syntrophobacter fumaroxidans*^35^. As for the *Candidatus Cloacamonas* species similar to the *acidaminovorans* strain (Fig. S4), potential links to IET have previously been drawn^13^. Its genome does contain putative membrane proteins and genes encoding for type IV pili such as PilB, PilC as well as PilT^13^,^36^ but no link to DIET has been reported yet.

The key species that could potentially be involved in DIET belongs to the *Geobacteraceae* family and was identified as *Geobacter hephaestius* (Fig. S4). Members of this family can use a variety of carbon sources including propionate^37^. Growth of *Geobacter* was affected by increasing conductivity, with its abundance more significant at higher Cl^−^ to TP ratio, a condition where both *Syntrophobacter* and *Candidatus Cloacamonas* sp were sensitive.

At this stage and under the current experimental conditions, the results obtained, including lack of response to improved cation motility (TP compared to Cl^-^ controls), and no substantial population of known electroactive organisms, does not support the hypothesis that DIET is a mode for propionate oxidation in this community, or alternatively, that DIET is not controlled by proton mobility. Further investigation under acclimatised conditions and in a known electroactive system are both areas which could further clarify controlling mechanisms.

## Methods

### Inoculum

Anaerobic granules grown on high levels of organics, including propionic acid^38^, were obtained from an active anaerobic digester (Fosters Breweries, Yatala, Queensland). These were separated into 20 g lots and enclosed into clean zip lock bags and crushed using a mortar and pestle. Granules were crushed (10–100 microns) in order to better visualise communities during FISH and avoid artefacts due to the deep biofilms, and performance was investigated against intact granules. All tests were carried out in triplicate to allow for the calculation of 95% confidence intervals in means (two-tailed t-test).

### Specific Methane Activity (SMA) test

SMA tests were set up in static 250 ml serum flasks, each of which contained 20 g of wet crushed granules (2.27 ± 0.04 g L^−1^ dry), 15 mM of propionic acid (1.1 g L^−1^), and saline solution phosphate buffer to potassium chloride ratio (TP:Cl^−^) of 1:0, 1:1, 2:1, 1:2 and 0:1, with molar concentration (Table S1) corresponding to the original effluent conductivity (1.5 mS cm^−1^-control), a 10X or a 30X strength and making up to a final volume of 100 ml with modified anaerobic CP media containing 1 ml NH_4_OH (10 g L^−1^), 1 ml MgCl.6H_2_O (8 g L^−1^), 1 ml of acid trace element solution (7.5 mM FeCl_2_.4H_2_O, 1 mM H_3_BO_3_, 0.5 mM MnCl_2_, 0.5 mM CoCl_2_, 0.5 mM ZnCl_2_, 0.1 mM NiCl_2_.6H_2_O and 50 mM HCl), 1 ml alkaline trace element (0.1 mM Na_2_SeO_3_. 5H_2_O, 0.1 mM Na_2_WO_4_, 0.1 mM Na_2_MoO_4_ and 10 mM NaOH), 1% v/v of filter sterilised mixture of vitamin solution (2 g L^−1^ biotin, 2 g L^−1^ folic acid, 10 g L^−1^ pyridoxine acid, 5 g L^−1^ riboflavin, 5 g L^−1^ thiamine, 0.1 g L^−1^ cyanocobalamine, 5 g L^−1^ nicotinic acid, 5 g L^−1^ P-aminobenzoic acid, 5 g L^−1^ lipoic acid and 5 g L^−1^ pantothenic acid) and 8 g L^−1^ CaCl_2_^39^. The 150 ml headspace was flushed with pure N_2_ gas, closed tightly and incubated at 37 °C. Batch analysis using NaCl instead of KCl was also carried out to assess relative inhibition effects (Fig. S1).

### Chemical analysis

#### Biogas analysis

Methane (CH_4_), hydrogen (H_2_) and carbon dioxide (CO_2_) were analysed at regular intervals by Gas Chromatography (GC) analysis. Using a 10 ml glass syringe, 5 ml of sampled headspace was analysed in a gas chromatograph (Perkin Elmer, Autosystem) with a thermo conductivity detector (GC-TCD) at 100 °C. a 99.99% purity Nitrogen (N_2_) gas was used as the carrier through a column (Alltech, 8011/2) of 50 m to 320 μm diameter. Results are reported as gCOD L^−1^ of CH_4_ produced over time. For every mole of propionic acid in the batch at initial conditions 2.75 mole of CH_4_ is expected with 0.75 moles produced from hydrogenotrophic methanogenesis and 2 moles from acetoclastic methanogenesis (derived from Eq. 1).

#### VFA analysis

Liquid samples, (3-4 ml), were taken at the same intervals as the gas samples and were filter-sterilised using 0.22 μm pore size Millipore^TM^ membrane cartridge filters. A 1:4 dilution was applied to the first 4 samples while the other samples remained undiluted. A 1% v/v of sodium azide (10% stock solution) was added to each vial before analysis by an HP Agilent high pressure liquid chromatography (HPLC) system, adapted with an inline degasser and a variable wavelength detector (VWD), at a flow rate of 0.0.8 ml min^−1^ and detection wavelength at 210 nm. In an attempt to achieve better separation, this analysis was carried out using a Carbo Sep Coregel-87H Guard Column, size 200 × 300 mm (Part No: ICE-99-9861) at a temperature of 65 °C.

### Modelling and parameter estimation

The maximum specific rate of the reaction (r_max_), half saturation coefficient (K_S_) and time delay (t_delay_) in substrate conversion were estimated through fitting of a Monod model (Eq. 4) to cumulative methane (CH_4_) production and substrate utilisation (equal weighting as COD). The model was implemented in Aquasim 2.1d^40^. Parameters were estimated by optimising the model to data using Aquasim’s secant (gradient search) method, with parameter uncertainty calculated from a two-tailed t-test on the linear estimate of the standard error calculated from the parameter Fisher information matrix^41^


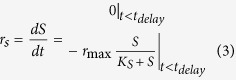


where r = specific rate of reaction

r_max_ = the maximum specific rate

S = concentration of substrate for growth

K_S_ = substrate half saturation coefficient

### Correlation analysis and p-values

Correlation between model parameters, operational conditions, and microbial factors has been assessed using the Pearson correlation coefficient (*r*) and the p-value for linear model vs H_0_ data = average.

### Changes in biomass as increase in Volatile Solids

Volatile solids (VS) were measured using the crucible based drying (105 °C) and volatilisation (650 °C) method described by Franson *et al.* (2005)^42^. The data (in grams) thus obtained were used to calculate Total Solids (TS) (Eq. 4) and VS (Eq. 5) values bases on the following equations:


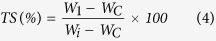



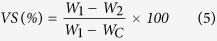


### Fluorescent *in situ* Hybridisation (FISH)

Samples were fixed in 4% paraformaldehyde solution (PFA) in a 1:2 ratio^43^. A 10 μl volume of the fixed sample was mounted onto a gelatine coated microscopic slide and hybridised with selected oligonucleotide probes; Syn835 for *Syntrophobacter* sp (5′-gcaggaatgagtacccgc-3′)^44^, EUB mix (consisting of EUB338 I-5′-tcgcaccgtggccgacacctag-3′, EUB338 II-5′-tcgcaccgtggccgacacctagc-3′ and EUB338 III-5′-tcgcaccgtggccgacacctagc-3′)^45^ for all other bacteria, MX825 (5′-tcgcaccgtggccgacacctagc-3′)^46^ for *Methanosaeta* sp and ARC915 (5′-tgctcccccgccaattcct-3′)^47^ for all archaea. The slides were examined under the confocal scanning laser microscopy (CSLM) microscope (Zeiss LSM 512) using Argon/2 (FITC), HeNe1 (Cy3) and HeNe2 (Cy5) lasers.

## 16S rRNA gene pyrosequencing

Total DNA was extracted from the granules prior and at the end of the batches using the PowerSoil® DNA Isolation Kit (MB 12888-MO BIO Laboratories, Inc.) with the following procedure; 2 g of sample was added to the PowerBead tubes. The samples were mechanically disrupted by bead beating at high speed for 60 seconds (Biopsec mini bead beater^TM^) followed by vortexing for another 60 seconds. DNA concentration and quality were determined by nanodrop (ND-1000) before being used for 16s rRNA gene amplification through polymerase chain reaction (PCR). Amplification of the 16S rRNA gene region was verified using primer set of pyroL926F (5′-aaactyaaakgaattgacgg-3′) and pyroL1392R (5′-acgggcggtgtgtrc-3′) before submission to the Australian Centre for Ecogenomics (ACE, University of Queensland) for gene pyrotag sequencing on a 454 sequencing technology platform (Roche, USA) with the same primers.

### Analysis of pyrosequencing data

Pyrosequencing data was grouped in operational taxonomic units (OTUs) at 97% similarity and aligned with the 16S rRNA identified sequences in the Greengenes database via the Quantitative Insights Into Microbial Ecology (QIIME) software package. These were further refined using the ACACIA software^48^.

### Principal Component Analysis (PCA)

Normalised OTU values were Hellinger transformed to enhance subdominants^24^. A principal component analysis (PCA), based on eigenvector multivariate analysis, was done using the vegan functions RDA in RStudio (R-project for statistical computing-R Foundation) and a Biplot used to visualise data.

### Phylogenetic analysis of pyrosequencing data

Cluster identity at species level was assessed using phylogenetic analysis. The 16S rRNA sequence from the 10 dominant clusters were aligned in MEGA 5.10 (molecular evolutionary genetics analysis – www.megasoftware.net) imported into the ARB software. These were first aligned with existing sequences from the database before generating a normalised neighbour joining tree at 10 000 iterations.

## Additional Information

**How to cite this article**: Juste-Poinapen, N. M. S. *et al.* Evaluating the potential impact of proton carriers on syntrophic propionate oxidation. *Sci. Rep.* **5**, 18364; doi: 10.1038/srep18364 (2015).

## Supplementary Material

Supplementary Information

## References

[b1] Thauer R. K., Jungermann K. & Decker K. Energy conservation in chemotrophic anaerobic bacteria. Microbiol. Mol. Biol. Rev. 41, 100–180 (1977). 10.1128/br.41.1.100-180.1977 PMC413997 860983

[b2] Stams A. J. M. & Plugge C. M. Electron transfer in syntrophic communities of anaerobic bacteria and archaea. Nat Rev Micro 7, 568–577 (2009). 10.1038/nrmicro2166 19609258

[b3] Lovley D. R. The microbe electric: conversion of organic matter to electricity. Current Opinion in Biotechnology 19, 564–571 (2008). 19000760 10.1016/j.copbio.2008.10.005

[b4] Boone D. R. & Bryant M. P. Propionate-Degrading Bacterium, *Syntrophobacter wolinii* sp. nov. gen. nov., from Methanogenic Ecosystems. Appl. Environ. Microbiol. 40, 626–632 (1980). 16345640 10.1128/aem.40.3.626-632.1980 PMC291629

[b5] Chen S., Liu X. & Dong X. *Syntrophobacter* sulfatireducens sp. nov., a novel syntrophic, propionate-oxidizing bacterium isolated from UASB reactors. International Journal of Systematic and Evolutionary Microbiology 55, 1319–1324 (2005). 15879275 10.1099/ijs.0.63565-0

[b6] de Bok F. A. M., Roze E. H. A. & Stams A. J. M. Hydrogenases and formate dehydrogenases of *Syntrophobacter fumaroxidans*. Antonie van Leeuwenhoek 81, 283–291 (2002). 12448727 10.1023/a:1020539323190

[b7] Imachi H. *et al.* Pelotomaculum propionicicum sp. nov., an anaerobic, mesophilic, obligately syntrophic, propionate-oxidizing bacterium. Int J Syst Evol Microbiol 57, 1487–1492 (2007). 17625181 10.1099/ijs.0.64925-0

[b8] Liu Y., Balkwill D. L., Aldrich H. C., Drake G. R. & Boone D. R. Characterization of the anaerobic propionate-degrading syntrophs *Smithella propionica* gen. nov., sp. nov. and *Syntrophobacter wolinii*. International Journal of Systematic Bacteriology 49, 545–556 (1999). 10319475 10.1099/00207713-49-2-545

[b9] Pelletier E. *et al.* “*Candidatus Cloacamonas Acidaminovorans*”: Genome Sequence Reconstruction Provides Glimpse of a New Bacterial Division. Journal of Bacteriology 190, 2572–2579 (2008). 18245282 10.1128/JB.01248-07 PMC2293186

[b10] Sieber J. R., McInerney M. J. & Gunsalus R. P. Genomic Insights into Syntrophy: The Paradigm for Anaerobic Metabolic Cooperation. Annual Review of Microbiology 66, 429–452 (2012). 10.1146/annurev-micro-090110-102844 22803797

[b11] Decker K., Jungermann K. & Thauer R. K. Energy Production in Anaerobic Organisms. Angewandte Chemie International Edition in English 9, 138–158 (1970). 10.1002/anie.197001381 4984685

[b12] Liu Y., Balkwill D. L., Aldrich H. C., Drake G. R. & Boone D. R. Characterisation of the anaerobic propionate-degrading syntrophs *Smithella propionica* gen. nov. sp. nov. and *Syntrophobacter wolinii*. Int J Syst Evol Microbiol. 545–556 (1995). 10.1099/00207713-49-2-545 10319475

[b13] Pelletier E. *et al.* “*Candidatus Cloacamonas Acidaminovorans*”: Genome Sequence Reconstruction Provides a First Glimpse of a New Bacterial Division. J. Bacteriol. 190, 2572–2579 (2008). 18245282 10.1128/JB.01248-07 PMC2293186

[b14] de Bok F. A. M., Plugge C. M. & Stams A. J. M. Interspecies electron transfer in methanogenic propionate degrading consortia. Water Research 38, 1368–1375 (2004). 15016514 10.1016/j.watres.2003.11.028

[b15] Deppenmeier U., Müller V. & Gottschalk G. Pathways of energy conservation in methanogenic archaea. Archives of Microbiology 165, 149–163 (1996).

[b16] Thauer R. K. Biochemistry of methanogenesis: a tribute to Marjory Stephenson:1998 Marjory Stephenson Prize Lecture. Microbiology 144, 2377–2406 (1998). 9782487 10.1099/00221287-144-9-2377

[b17] Schlegel K. & Müller V. Evolution of Na (+) and H (+) bioenergetics in methanogenic archaea. Biochemical Society Transactions 41, 421 (2013). 23356322 10.1042/BST20120294

[b18] Dong X. & Stams A. J. M. Evidence for H2 and formate formation during syntrophic butyrate and propionate degradation. Anaerobe 1, 35–39 (1995). 16887505 10.1016/s1075-9964(95)80405-6

[b19] Batstone D. J., Picioreanu C. & van Loosdrecht M. C. M. Multidimensional modelling to investigate interspecies hydrogen transfer in anaerobic biofilms. Water Research 40, 3099–3108 (2006). 16901527 10.1016/j.watres.2006.06.014

[b20] Summers Z. M. *et al.* Direct Exchange of Electrons Within Aggregates of an Evolved Syntrophic Coculture of Anaerobic Bacteria. Science 330, 1413–1415 (2010). 21127257 10.1126/science.1196526

[b21] Morita M. *et al.* Potential for Direct Interspecies Electron Transfer in Methanogenic Wastewater Digester Aggregates. mBio 2 (2011). 10.1128/mBio.00159-11 PMC3157894 21862629

[b22] Rotaru A.-E. *et al.* A new model for electron flow during anaerobic digestion: direct interspecies electron transfer to *Methanosaeta* for the reduction of carbon dioxide to methane. Energy & Environmental Science 7, 408–415 (2014).

[b23] Lovley D. Reach out and touch someone: potential impact of DIET (direct interspecies energy transfer) on anaerobic biogeochemistry, bioremediation, and bioenergy. Reviews in Environmental Science and Bio/Technology 10, 101–105 (2011).

[b24] Torres C. I. *et al.* A kinetic perspective on extracellular electron transfer by anode-respiring bacteria. FEMS Microbiology Reviews 34, 3–17 (2010). 19895647 10.1111/j.1574-6976.2009.00191.x

[b25] Engasser J.-M. & Wilhelm-Despres A.-M. The facilitation of proton transport by acids and bases. Chemical Engineering Science 35, 669–672 (1979).

[b26] Torres C. I., Kato Marcus A. & Rittmann B. E. Proton transport inside the biofilm limits electrical current generation by anode-respiring bacteria. Biotechnology and Bioengineering 100, 872–881 (2008). 18551519 10.1002/bit.21821

[b27] Nagarajan H. *et al.* Characterization and modelling of interspecies electron transfer mechanisms and microbial community dynamics of a syntrophic association. Nature Communications 4 (2013). 10.1038/ncomms3809 24264237

[b28] Solera R., Romero L. I. & Sales D. Determination of the Microbial Population in Thermophilic Anaerobic Reactor: Comparative Analysis by Different Counting Methods. Anaerobe 7, 79–86 (2001).

[b29] Buswell A. M. Fundamentals of Anaerobic Treatment of Organic Wastes. Sewage and Industrial Wastes 29, 717 (1957).

[b30] Schlenz H. E. Important Considerations in Sludge Digestion. Sewage Works Jour. 19, 19 (1947). 20295641

[b31] McCarty P. L. & McKinney R. E. Volatile Acid Toxicity in Anaerobic Digestion. Journal (Water Pollution Control Federation) 33, 223–232 (1961).

[b32] Oren A. (ed.) Adaptation of halophilic archaea to life at high salt concentrations (Springer: Dordrecht, The Netherlands, 2004).

[b33] Galinski E. A. In Advances in Microbial Physiology (ed. Poole R. K.) 273–328 (Academic Press, 1995).

[b34] Imachi H. *et al.* *Methanolinea* tarda gen. nov., sp. nov., a methane-producing archaeon isolated from a methanogenic digester sludge. International Journal of Systematic and Evolutionary Microbiology 58, 294–301 (2008). 18175725 10.1099/ijs.0.65394-0

[b35] Plugge C. M. *et al.* Complete genome sequence of *Syntrophobacter fumaroxidans* strain (MPOBT). Standard in Genomic Sciences 7, 91–106 (2012). 10.4056/sigs.2996379 PMC3570798 23450070

[b36] Hugenholtz P. & Kyrpides N. C. A changing of the guard. Environmental Microbiology 11, 551–553 (2009). 19278443 10.1111/j.1462-2920.2009.01888.x

[b37] Gan Y., Qiu Q., Liu P., Rui J. & Lu Y. Syntrophic Oxidation of Propionate in Rice Field Soil at 15 and 30 °C under Methanogenic Conditions. Applied and Environmental Microbiology 78, 4923–4932 (2012). 22582054 10.1128/AEM.00688-12 PMC3416378

[b38] Batstone D. J. & Keller J. Variation of bulk properties of anaerobic granules with wastewater type. Water Research 35, 1723–1729 (2001). 11329674 10.1016/s0043-1354(00)00446-2

[b39] Plugge C. M. In Methods in Enzymology (ed. Jared R. L.) 3–16 (Academic Press, 2005).16260282

[b40] Reichert P. Aquasim-A tool for simulation and data-analysis of aquatic systems. Water Science and Technology 30, 21–30 (1994).

[b41] Ralston M. L. & Jennrich R. I. Dud, A Derivative-Free Algorithm for Nonlinear Least Squares. Technometrics 20, 7–14 (1978).

[b42] Franson M. A. H., Eaton A. D., Association. A. P. H., Association. A. W. W. & Federation W. E. Standard methods for the examination of water & wastewater (American Public Health Association, Washington, DC, 2005).

[b43] Hugenholtz P., Tyson G. W., Webb R. I. & Blackall L. L. Investigation of candidate division TM7, a recently recognised major lineage of the domain bacteria with no known pure-culture representatives. Appl Environ Microbiol 67, 411–419 (2001). 11133473 10.1128/AEM.67.1.411-419.2001 PMC92593

[b44] Fernández N., Díaz E., Amils R. & Sanz J. Analysis of Microbial Community during Biofilm Development in an Anaerobic Wastewater Treatment Reactor. Microbial Ecology 56, 121–132 (2008). 18034358 10.1007/s00248-007-9330-2

[b45] Amann R. I. *et al.* Combination of 16S rRNA-targeted oligonucleotide probes with flow cytometry for analyzing mixed microbial populations. Applied and Environmental Microbiology 56, 1919–1925 (1990). 2200342 10.1128/aem.56.6.1919-1925.1990 PMC184531

[b46] Raskin L., Stromley J. M., Rittmann B. E. & Stahl D. A. Group-specific 16S rRNA hybridization probes to describe natural communities of methanogens. Applied and Environmental Microbiology 60, 1232–1240 (1994). 7517128 10.1128/aem.60.4.1232-1240.1994 PMC201464

[b47] Yanagita K. *et al.* Phylogenetic analysis of methanogens in sheep rumen ecosystem and detection of Methanomicrobium mobile By fluorescence *in situ* hybridization. Bioscience, biotechnology, and biochemistry 64, 1737–1742 (2000). 10.1271/bbb.64.1737 10993166

[b48] Bragg L. M., Stone G., Butler M. K., Hugenholtz P. & Tyson G. W. Shining a Light on Dark Sequencing: Characterising Errors in Ion Torrent PGM Data. PLoS Computational Biology 9, e1003031 (2013). 23592973 10.1371/journal.pcbi.1003031 PMC3623719

