# Supplementary material for: Evaluating the potential impact of proton carriers on syntrophic propionate oxidation
Source: Sci Rep. 2015 Dec 16;5:18364. doi: 10.1038/srep18364 (PMC4680937; doi:10.1038/srep18364)
Supplement: Supplementary Information [file srep18364-s1.pdf]

Supplemental Materials for:

Evaluating the potential impact of proton carriers on syntrophic propionate oxidation

Authors: Natacha M. S. Juste-Poinapen, Mark S. Turner, Korneel Rabaey, Bernardino Virdis, and Damien J. Batstone

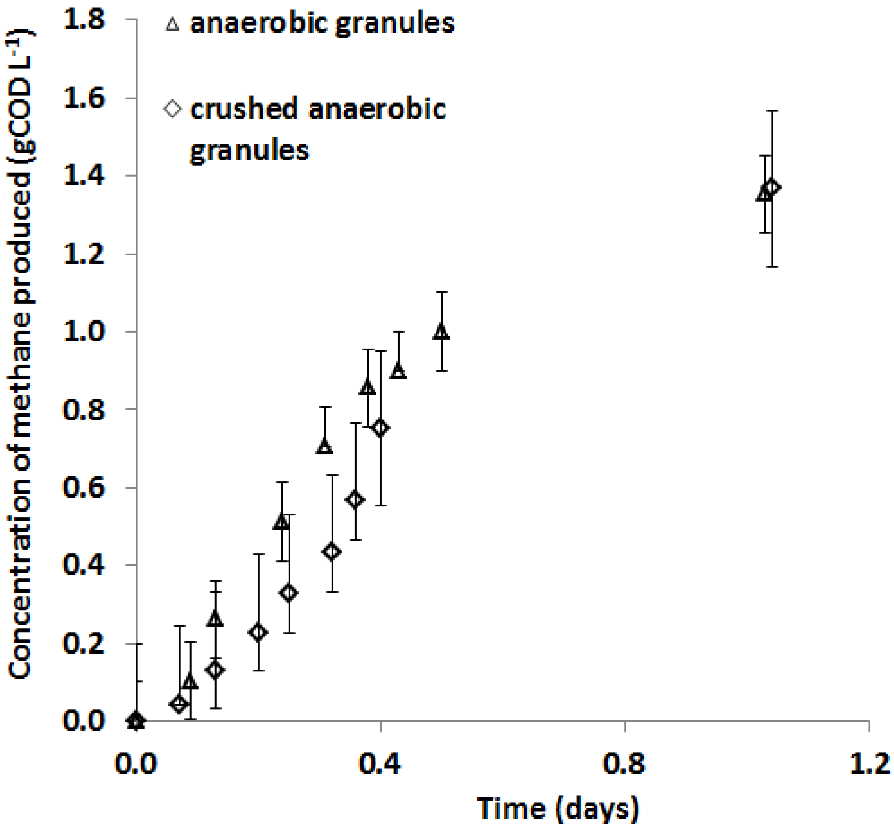

Figure S1: Cumulative methane production measured to compare the performance of intact anaerobic granules to crushed anaerobic granules during syntrophic propionic acid oxidation

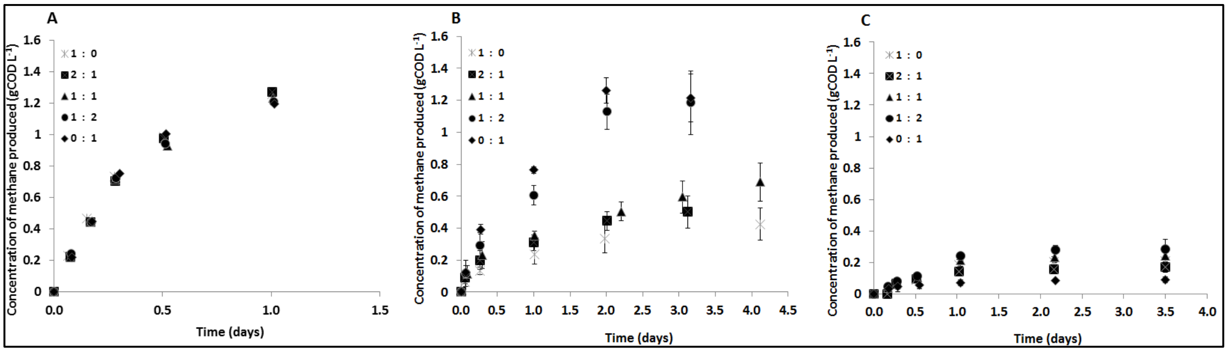

Figure S2: Growth curves with methane produced (gCOD L^{-1}) during the oxidation of propionic acid with NaCl, instead of KCl as the chloride compound

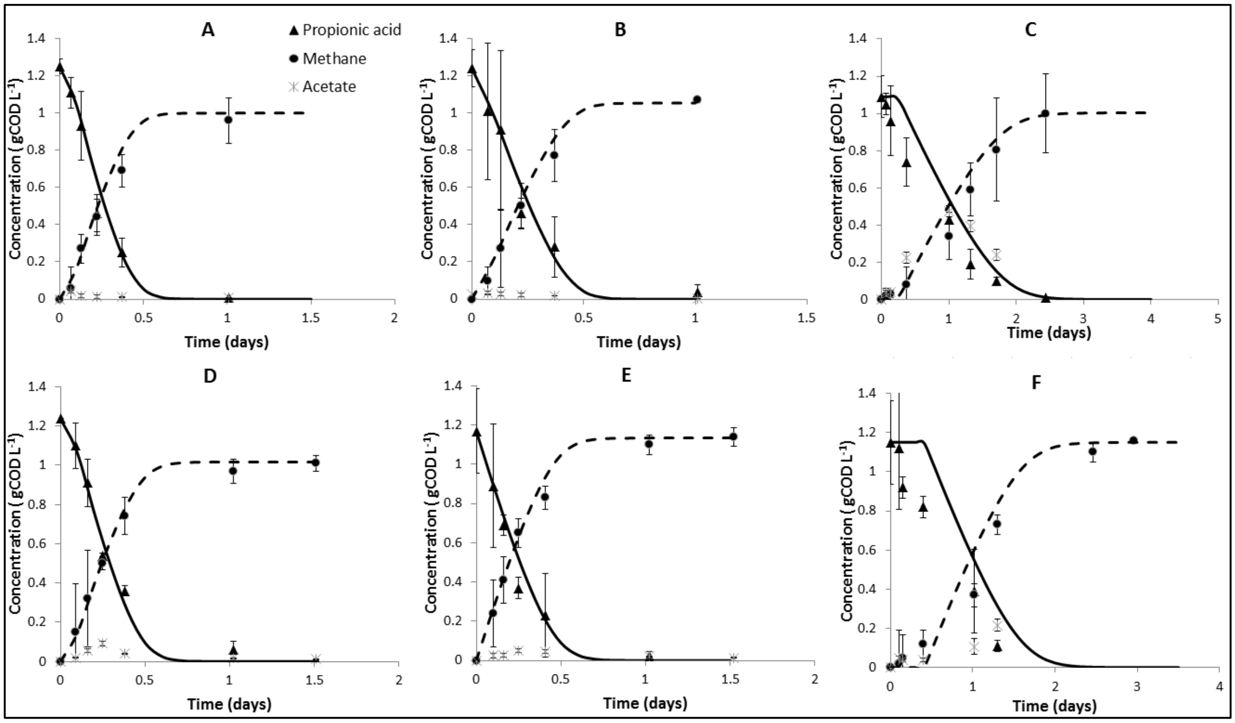

Figure S3: Methane production (dots) and propionic acid utilisation (triangles) data fitted into growth curves for assays with 2TP: Cl^- and TP: $2Cl^-$ at initial, 10X and 30X conductivity. The plateau at the beginning showing the time delay (t_{delay}) encountered at highest conductivity. Error bars represent 95% confidence based on triplicate analyses

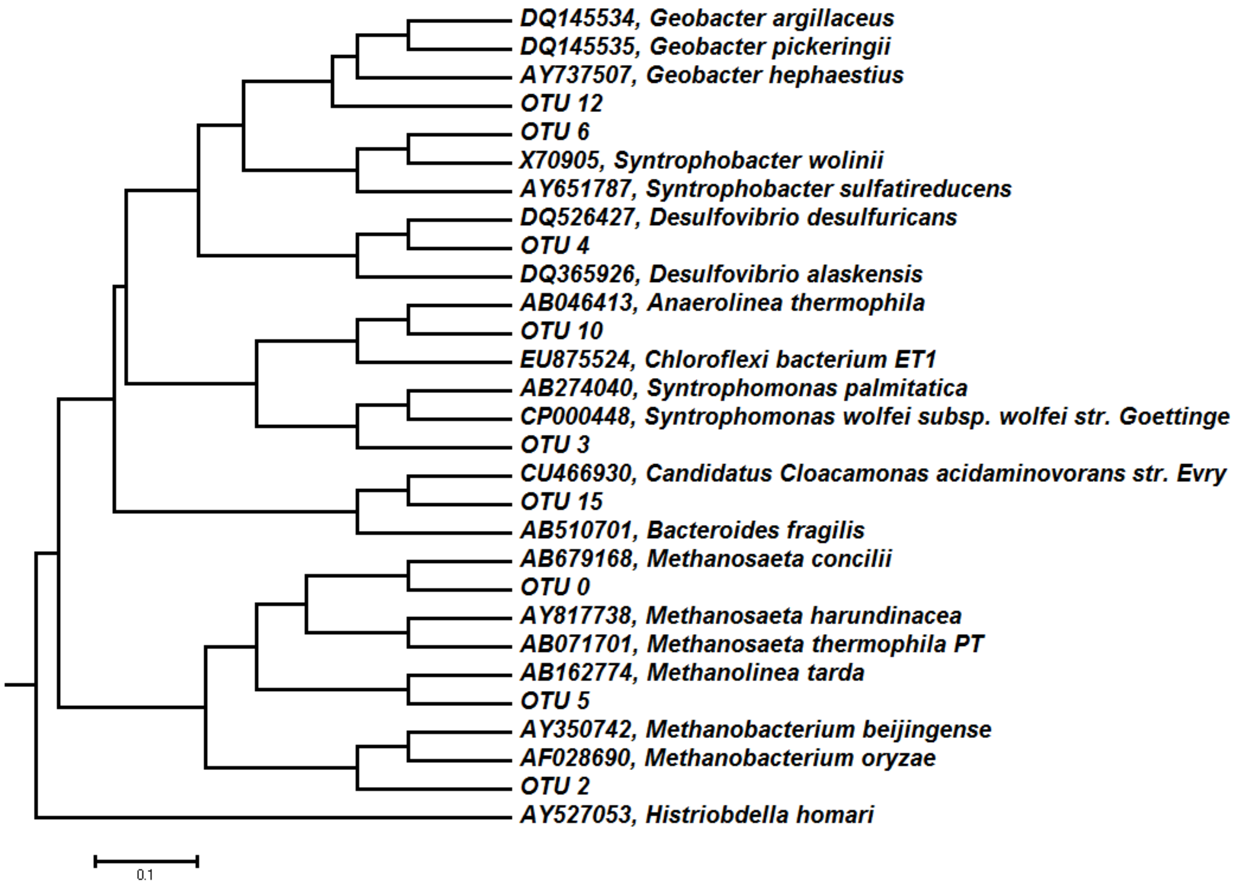

Figure S4: Neighbour-joining phylogenetic tree based on partial 16S rRNA gene sequences representing the nine most dominant species revealed during pyrosequencing analysis of samples treated at various TP : Cl^- ratio and increasing conductivity combinations. The tree was constructed with 1000 iterations and the bar indicates 10% sequence divergence

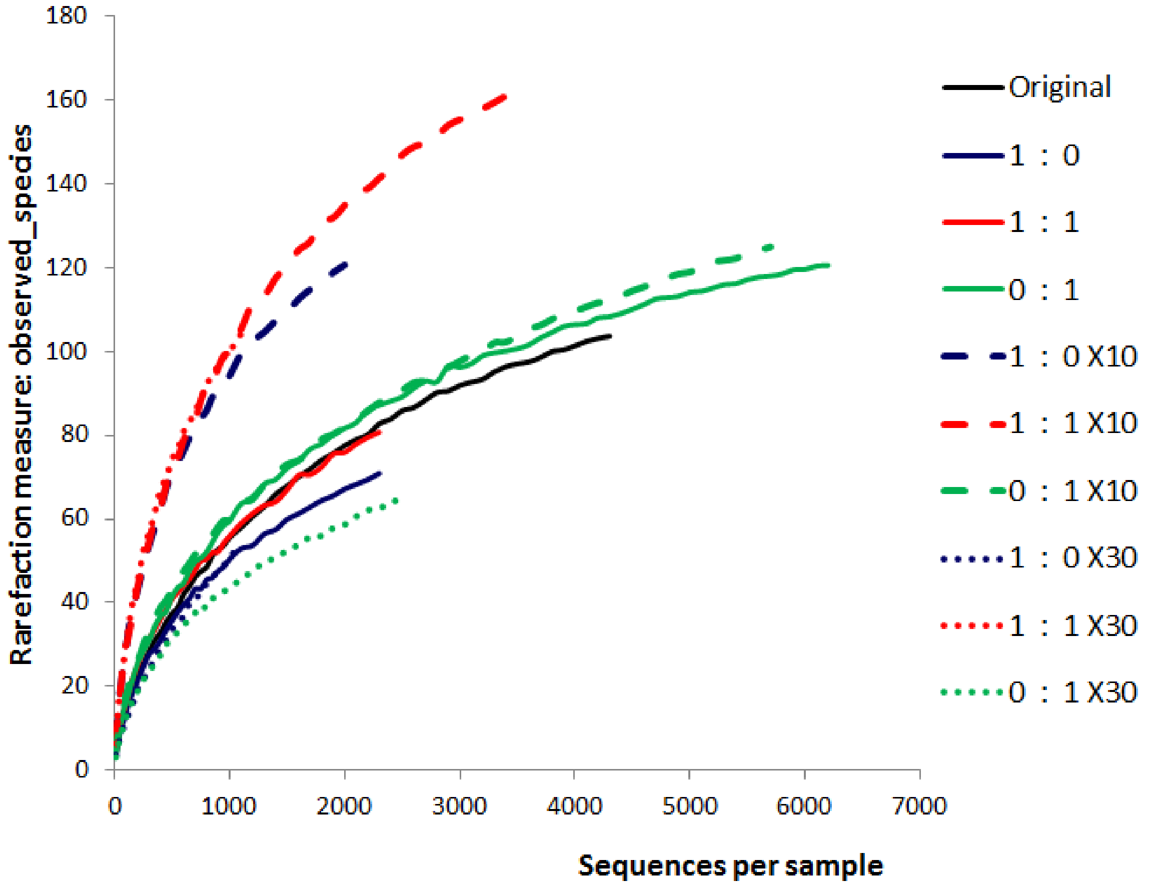

Figure S5: Diversity analysis using the rarefaction measure based on the number of sequences and the number of observed species for each sample. As compared to the original sample (solid, black) assays with more diversity in descending order were TP: Cl^- at 10X (broken, red), TP: Cl^- at 30X (dotted, red), TP only at 10X (broken, blue), Cl^- only at 10X (broken, green) and Cl^- only at 1X conductivity. The least diverse assays in descending order were TP: Cl^- at 1X (solid, red), TP only at 1X (solid, blue), TP only at30X (dotted, blue) and Cl^- only at 30X (dotted, green) conductivity

Conductivity	Concentration at different TP: Cl^- ratio (mM)				
	**1:0**	**2:1**	**1:1**	**2:1**	**0:1**
**1X (Control)**	1	0.6:0.3	0.5:0.5	0.3:0.6	0.9
**10X**	11.5	7.3:3.4	5.3:5.4	3.5:7.0	10.4
**30X**	36.5	23.7:11.8	17.5:17.5	11.3:22.7	33.5

Table S1: Summary of the different molar concentration of total phosphate (TP) and chloride ions in each treatment

Conductivity	X1	X10	X30
	VS (g L^{-1}) ± CI	VS (g L^{-1}) ± CI	VS (g L^{-1}) ± CI
1:0	2.30 ± 0.62	2.68 ± 0.07	2.45 ± 1.84
2:1	2.60 ± 0.25	2.49 ± 0.07	2.34 ± 1.28
1:1	2.58 ± 0.79	2.71 ± 0.09	2.52 ± 0.54
1:2	2.51 ± 1.65	2.65 ± 0.71	2.42 ±1.59
0:1	2.44 ± 0.75	2.54 ± 1.57	2.30 ± 0.25

Table S2: Average Volatile Solids (VS) concentration in each treatment corresponding to increases in biomass, for 3 biological repeats of each experiment. All results are presented with ± 95% confidence intervals

Taxonomy	Microorganisms	Normalised OTU (%)									
		Samples									
		**Original**	**1 : 0**	**1 : 1**	**0 : 1**	**1 : 0 (10X)**	**1 : 1 (10X)**	**0 : 1 (10X)**	**1 : 0 (30X)**	**1 : 1 (30X)**	**0 : 1 (30X)**
Bacteria	*Syntrophomonas*	4.40	5.73	5.60	6.00	1.47	1.73	4.93	7.33	2.00	3.60
	SHA-114	3.73	0.27	0.80	0.67	0.93	1.73	3.07	2.13	0.40	3.33
	Bacteroidales	2.53	2.80	1.73	2.13	0.93	0.93	1.73	2.00	0.93	2.53
	Syntrophobacter	1.20	1.87	1.07	0.93	3.33	2.93	2.53	0.67	2.27	1.33
	Anaerolinea	1.07	0.27	0.00	0.13	0.00	0.00	0.13	0.00	0.00	0.00
	GCA004	0.93	1.47	0.80	1.33	0.93	1.07	0.80	1.07	0.53	1.47
	Candidatus Cloacamonas	0.67	1.20	0.53	0.67	0.80	2.53	1.33	0.53	1.73	0.40
	Geobacteraceae	0.53	1.07	0.27	2.13	0.80	0.67	1.87	1.07	0.93	0.53
	Longilinea	0.53	0.27	0.00	0.27	0.67	0.27	0.00	0.13	0.40	0.13
	Syntrophorhabdaceae	0.40	0.00	0.13	0.13	0.00	0.00	0.13	0.13	0.00	0.13
	Anaerolinea	0.40	0.27	0.13	0.40	0.00	0.27	0.27	0.13	0.00	0.40
	SHA-114	0.27	0.13	1.07	0.40	0.53	0.80	0.40	0.13	1.20	0.00
	Phycisphaerae	0.27	0.00	0.00	0.13	0.53	0.13	0.00	0.13	0.53	0.13
	Syntrophaceae	0.13	0.40	0.67	0.13	0.53	0.53	0.27	0.13	0.13	0.40
	TA06	0.13	0.40	0.53	0.27	0.80	0.67	0.00	0.13	0.53	0.13
	Desulfovibrio	0.13	0.13	0.53	0.13	0.93	0.13	0.00	0.53	0.53	0.27
	OPB11	0.13	0.00	0.00	0.13	0.13	0.13	0.27	0.13	0.13	0.00
	Acinetobacter	0.00	0.00	0.00	0.00	0.00	0.00	0.00	0.00	0.00	0.00
	Bacteroidales	0.00	0.53	0.27	0.00	0.13	0.27	0.13	0.27	0.27	0.00
	WCHB1-15	0.00	0.53	0.53	0.40	0.40	0.00	0.00	0.13	0.53	1.20
	Aeromonadales	0.00	0.00	0.00	0.00	0.00	0.13	0.00	0.00	0.00	0.00
	Acinetobacter	0.00	0.00	0.00	0.00	0.00	0.00	0.00	0.00	0.00	0.00
	SR1	0.00	0.13	0.53	0.13	0.00	0.13	0.00	0.00	0.53	0.27
Archaea	*Methanobacteriales*	44.13	8.27	9.07	12.00	24.40	19.47	10.80	38.27	34.13	13.33
	Methanosaeta	30.27	65.47	64.93	60.27	42.93	46.53	61.20	38.93	36.13	59.60
	Methanosaeta	0.80	0.40	0.40	0.13	0.00	0.27	0.53	0.27	0.00	0.27
	WCHD3-02	0.67	0.27	0.67	0.67	0.80	0.27	0.93	0.27	0.67	0.40
	Methanobacterium	0.53	0.40	0.00	0.00	0.27	0.13	0.00	0.27	0.00	0.53
	Methanobacterium	0.53	0.00	0.00	0.00	0.00	0.27	0.13	0.13	0.13	0.13
	Methanolinea	0.40	2.40	2.67	3.20	6.13	6.40	2.80	0.40	3.47	2.93
	Thermoprotei	0.40	0.13	0.13	0.13	0.67	0.53	0.40	0.13	0.00	0.13
	Methanosaeta	0.40	0.27	0.00	0.00	0.00	0.13	0.13	0.13	0.00	0.13
	Methanoregulaceae	0.27	0.53	0.80	1.20	2.67	3.47	0.80	0.40	3.73	0.67
	WCHD3-02	0.13	0.13	0.27	0.27	0.67	1.07	0.67	0.00	0.53	0.40

Table S3: Relative abundances of bacterial and archaeal taxa based on OTU data obtained at a 97% confidence threshold. Values represent percentages of domain bacteria and archaea after Hellinger transformation

Conductivity	Ratio		
	**1:0**	**1:1**	**0:1**
1X	2.03	2.48	2.59
10X	4.37	4.38	2.76
30X	2.20	4.30	1.93

Table S4: Comparison of the Shannon index for each assay carried out with TP only, TP: Cl^- and Cl^- at 1X, 10X and 30X conductivities. A higher value indicates a diverse and equally distributed community
